# Can COVID-Era Export Restrictions Be Deterred?

**DOI:** 10.1017/S0008423920000578

**Published:** 2020-06-10

**Authors:** Krzysztof Pelc

**Affiliations:** Department of Political Science, McGill University, 855 Sherbrooke St. W., Montreal, QC, H3A 2T7

## Abstract

The COVID-19 pandemic has led some 75 countries to restrict their exports of hundreds of essential products, ranging from antibiotics and face masks to medical ventilators. Since banning exports decreases global supply and leads to price surges on world markets, the cost of these measures may ultimately be counted in human lives.

The COVID-19 pandemic has led some 75 countries to restrict their exports of hundreds of essential products, ranging from antibiotics and face masks to medical ventilators. Since banning exports decreases global supply and leads to price surges on world markets, the cost of these measures may ultimately be counted in human lives.

To make matters worse, the international trade regime is ill-designed to deal with export restrictions. Since the beginning of the pandemic, trade experts have called for greater global cooperation (Beattie, 2020), yet such level-headed appeals ignore the long and unsuccessful history of attempts to discipline export restraints. Export restraints currently fall into something of a legal grey zone: they are nominally considered violations, but there are sufficient exceptions written into multilateral rules to render them permissible in any circumstances under which they may be needed, including the current pandemic. This is not happenstance. For the past 70 years of multilateral trade negotiations, there has been a widespread recognition that governments are unlikely to commit to meaningful discipline on export restraints, given the necessity to secure domestic supplies by any means necessary in times of crisis.

Given how lax international rules are, scholars have concluded that “the only real deterrent to export bans [during the pandemic] is the threat of foreign retaliation that cuts off access to indispensable imports” (Pauwelyn, 2020). Faith in the deterrent effect of retaliation is widely shared among trade experts, who draw on the experience of retaliatory patterns in import tariffs. The precedent of the Smoot–Hawley tariff of 1930 is often cited in making these claims. The most influential trade policy analysis since the start of the pandemic, by Baldwin and Evenett (2020: 7), thus concludes: “Just as the 1930s tariffs triggered demand-crushing tariff retaliation, today's export strictures risk triggering a retaliatory spiral that ultimately destroys supply. This is great folly.” Other trade experts have echoed these warnings. Chad Bown of the Petersen Institute has warned that “export restrictions could trigger a spiral of retaliation” (Bown, 2020: 32). And a group of World Bank economists have prophesied that “restrictions to exports are inherently beggar-thy-neighbour policies, which is why they induce retaliation rather than cooperation” (Espitia et al., 2020). The US Congressional Research Service, the World Trade Organization (WTO), and the International Monetary Fund (IMF) have similarly warned of spirals of retaliation in export restrictions (CRS, 2020).

Does retaliation actually take place, and can its threat effectively deter export restrictions during crises? The early evidence suggests the answer is no. We cannot rely on the threat of retaliation to deter most export controls, because the most flagrant users are also shielded by the very characteristics that render them prone to imposing export restraints in the first place. The empirical evidence suggests that the prospect of retaliation has played no role in the decision to restrict exports by the world's largest producers of essential medical goods.

## The Story Thus Far

**Figure 1:**
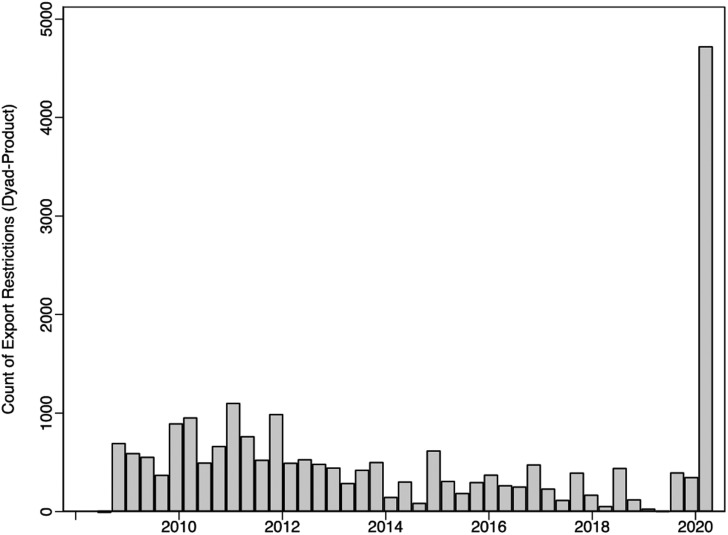
Export controls worldwide over time (monthly count).

Figure 1 provides a sense of the surge in export restrictions witnessed thus far during the COVID-19 pandemic. The data cover the period following the Great Recession up to May 2020 and come from Global Trade Alert (GTA), which currently comprises the most comprehensive available record of global trade barriers. The extent to which countries have rushed to restrict their exports is unprecedented. The closest analogue is found in the global food crises of 2008 and 2011, when a number of developing countries imposed restrictions on their grains exports, but these too pale in comparison with the current situation. The most affected product are pharmaceuticals, followed by medical ventilators, and various types of PPE.

While an unprecedented number of countries have moved to restrict their exports, a closer look reveals much variation between states in their reliance on such measures. Governments also appear to act strategically by shutting exports to some trade partners while excluding others. Countries that have restricted their exports during the COVID-19 era have targeted an average of 34 trading partners. The United States has thus banned exports of respirators and a range of PPE such as surgical masks and gloves, but it has excluded Mexico and Canada from this measure (see CRS, 2020: 2). The EU imposed a common licensing system on its exports after some of its member states imposed their own export controls on one another, affecting countries, like Italy, that had the greatest need for PPE. The EU then moved to exclude the EFTA countries (Iceland, Liechtenstein, Norway and Switzerland). Subsequent exemptions extended to the Balkan countries, four of which are candidates for EU membership (Blenkinsop, 2020; see also von der Burchard and Gray, 2020). India has done the most to restrict its exports, but it has also made exceptions for some neighbouring countries. After negotiations with the White House, India lifted its ban on hydroxychloroquine exports to the US, while maintaining other restrictions (Haidar, 2020). Such dyadic variation proves analytically valuable, providing unique insight into governments' decision-making.

**Figure 2:**
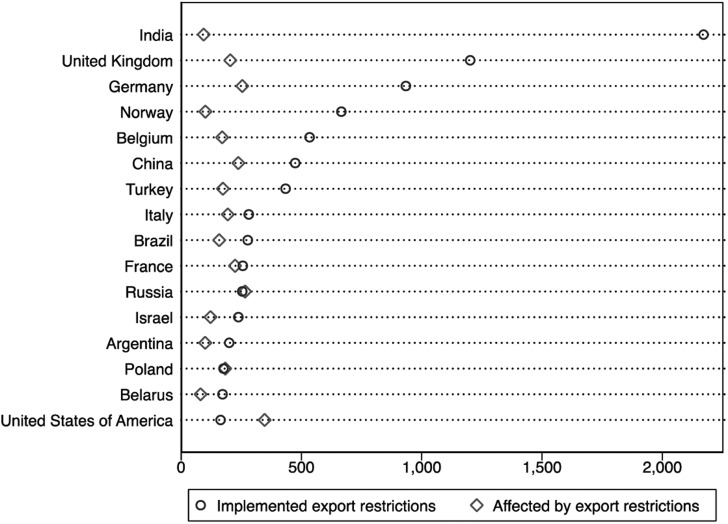
Top users of export restrictions during the COVID-19 pandemic.

Figure 2 lists the countries that have imposed the most restrictions on exports of essential medical goods, alongside the number of restrictions each has been targeted with. India has been by far the most aggressive country in banning exports during the pandemic. This is cause for concern, given how India now functions as a pharmacy to the world, supplying over half the world's vaccines and the greater portion of generic pharmaceuticals. By comparison, despite having decisively turned its back on global cooperation, the US has put in place far fewer export restrictions than European countries like the UK and Germany. Figure 2 also illustrates how little relation there is between a country's own behaviour and its treatment by others. As I argue next, this discrepancy, and what it suggests about the deterrent effect of retaliation, has to do with the unique features of export controls.

## 2.1 Legal Rules Over Export Controls

In the traditional mercantilist mindset that still permeates the global trade regime, countries usually seek to *promote* their exports. But this can change in the face of a demand shock like the current one, when many countries are in sudden need of the same products. States then tend to reduce their import tariffs and restrict their exports.

Article XI of the GATT nominally prohibits export restrictions, while making allowances for temporary measures meant to “prevent or relieve critical shortages” (WTO, 2020). In effect, all export restrictions that countries have imposed during the pandemic would likely fall under such exemptions. This lax treatment of export controls has been a feature of the trade regime since its beginnings. As a 1974 GATT Secretariat asked, “Why have there been so few concessions on export restrictions in the past despite the contracting parties' formal recognition that it would be desirable to negotiate them?” (GATT, 1974). The answer it provides is that states are “reluctant to commit themselves” to hard rules given the need to suspend them during emergencies.

Indeed, when the need for export restrictions does arise, the stakes are often life-and-death, and the resulting political pressure to act is far higher than in the case of most import tariffs. The necessity of keeping one's own healthcare workers supplied with face masks may dominate any concerns over global trade norms. The end result, of course, is that, globally, fewer healthcare workers ultimately get the face masks they require. Yet the nature of the underlying political incentives is such that we are unlikely to ever see effective formal disciplines that limit export restrictions. Recognizing this, scholars have hung their hopes on the risk of retaliation deterring the worst of export bans: if states sufficiently fear being cut off from imports of vital goods, they may keep from blocking their own exports. Is there evidence of such deterrence at work?

## Can Retaliation Function as a Deterrent?

Retaliation is always costly, but it can make strategic sense if it affects trade partners’ behaviour. Much of postwar trade cooperation rests on the implicit threat of retaliatory import tariffs. The classic reference in this respect is the Smoot–Hawley tariff, imposed by the US in 1930, which led over 60 countries to retaliate against the US, even as the world was in the grips of economic depression. These included close allies, like Canada, and small countries with no market power, like Switzerland.^1^ Since then, the threat of retaliatory import tariffs has been instrumental in preserving multilateral cooperation even in the midst of hard times, such as during the Great Recession (Davis and Pelc, 2017).

But things may look different with export restrictions: the same reason why banning exports may become politically imperative also makes it impossible for resource-dependent countries to impose their own measures in retaliation. This is why, as Figure 2 shows, India has massively restricted its exports, yet its trade partners have come short of retaliating in kind. Meanwhile, a country like Canada, which is highly dependent on its imports of PPE and pharmaceuticals, and lacks domestic manufacturing capacity in both (Lipkus, 2020), simply cannot afford to restrict its exports, least of all to India, on which it relies for a range of pharmaceuticals. Countries’ unequal export profiles thus translate into differences in market power.

Does this phenomenon extend past the extreme case of India? To find out, I construct a dyadic dataset of all export controls registered by Global Trade Alert from the start of 2020 to the end of May 2020. The unit of analysis is the dyad-product-measure: each observation corresponds to a distinct export restriction imposed by country *i* on country *j* over a six-digit HS product *x*.^2^ The data cover 10,261 such measures that were imposed by 75 different countries on 157 trade partners. I purposefully treat all measures restricting exports alike: I thus do not distinguish the type of measure^3^ nor weigh by the volume of affected trade, both of which could be further accounted for in future work.

The sample is made up of all dyads*_ij_*, where country *i* has restricted its exports at any point in 2020. I thus estimate each country *i*'s behavior with each trade partner *j* using a Poisson count model with errors clustered on the dyad. This amounts to asking the question: among countries that have restricted their exports against anyone, have trade partners responded in kind? I estimate the effect of the trade partner's own export restraints, the GDP, GDP per capita, and import dependence on the 10 most restricted medical goods since the start of the pandemic, for each country in a given dyad. I then also substitute the country-level variables with country fixed effects, first for the home (implementing) country and then for the trade partner (targeted country). The data are cross-sectional: there is no time dimension, given how all these restrictions were put in place over a short span of time. Future work, however, might do more to examine potential tit-for-tat patterns by testing whether countries grew more likely to impose export restrictions after being targeted themselves.

The findings, shown in Table 1, should serve as a warning to those hoping that export restraints will follow a retaliatory pattern that may dissuade countries from putting restrictions in place. Looking at dyadic country data since the start of the pandemic, retaliation appears to play no role in deterring states from imposing restrictions on their most vital pandemic exports. That is, the biggest users of export restrictions do not themselves face more export restrictions from the countries they target, as common expectations about retaliation would lead us to expect. If anything, the relationship between a country's export restrictions and its trade partner's response is consistently negative, though this relationship never approaches statistical significance.

**Table 1:** Determinants of dyadic export restrictions

	(1)	(2)	(3)	(4)
Trade partner export restrictions	–0.01	–0.01	–0.00	–0.00
	(0.01)	(0.01)	(0.00)	(0.01)
Home GDP (log)	0.68***	0.86***		0.86***
	(0.02)	(0.05)		(0.05)
Home GDP/cap (log)	–0.23***	–0.06		–0.08
	(0.04)	(0.05)		(0.05)
Trade partner GDP (log)	0.40***	0.17***	0.15***	
	(0.02)	(0.04)	(0.03)	
Trade partner GDP/cap (log)	0.10***	–0.00	0.00	
	(0.03)	(0.03)	(0.02)	
Home imports of pandemic products (log)		–0.29***		–0.29***
		(0.07)		(0.07)
Trade partner imports of pandemic products (log)		0.29***	0.30***	
		(0.05)	(0.03)	
Constant	–27.60***	–26.77***	–27.67	–22.40***
	(0.71)	(0.94)	(.)	(1.19)
Home country fixed effects			Yes	
Trade partner country fixed effects				Yes
Observations	11174	8610	9230	10990

Note: Poisson count model estimates. Dependent variable is frequency of export restrictions by country-dyad.

Robust standard errors clustered on shared dyad in parentheses.

*p < 0.10, **p < 0.05, ***p < 0.01.

The best country-level predictor of who gets hit with export restraints is their total demand for essential goods: the more “pandemic goods” a country imports, the less likely it is to be able to procure essential medical goods from its trade partners. The US, which has been hit with more export restrictions than any other country, may be the case in point. That those in greatest need may be least able to count on their trade partners is plainly worrisome.

The warnings about spirals of retaliation in export bans thus miss part of the story. While the fear of retaliation is in fact internalized by import-dependent countries like Canada, those countries, like India, that have the most to gain domestically from restricting their exports are also impervious to retaliation. Being a major supplier of essential goods affords them a degree of impunity.

## What Can Be Done?

If retaliation is unlikely to deter export restrictions by the largest producers of vital goods, what are we left with? A widespread reflex among governments has been to move toward autarky. A number of firms have stepped up to provide vital goods for their domestic markets: for instance, Bauer Canada, which makes hockey gear, has pivoted to manufacturing face shields for medical use, and the Quebec government has purchased 300,000 of these. What is often left unsaid is that at a price of $6 apiece, these face shields cost around five times more than the equivalent Chinese-made product (O'Connor, 2020). This underscores how any effective increase in the supply of vital goods has little choice but to harness global supply chains if it is to be effective. The challenge is to utilize the productive capacity of global economic integration in the fight against the pandemic while injecting sufficient “slack” into supply networks to pre-empt a proliferation of barriers in the wake of shocks.

In related work, I show that one significant factor that increases the odds of export restrictions is market consolidation (Pelc, 2020). Specifically, when the production of a good is highly concentrated across countries, export controls during crises appear more likely, all else equal. This augurs poorly for the current situation, given recent consolidation in the pharmaceutical industry, as seen in India's market share growth in the last two decades. Yet this link between export controls and market concentration also provides a possible direction for policy change.

A coordinated global initiative to limit market concentration in essential goods may be one effective means of preventing export bans during collective crises. The objective of such a scheme would be to identify the best alternative producers for goods judged essential and for which market supply is highly concentrated, and to allow governments to offer these producers import protection. Rather than pushing all countries to rely on their own domestic producers in a costly search for self-sufficiency, this would amount to seeking the next most efficient marginal producer worldwide. The WTO safeguard, which is designed to deal with sudden import surges, might provide a template. Paradoxically, export restrictions may themselves lead to such a broadening of production across countries, as new suppliers in countries that do not restrict exports rise to meet global demand, but they do so at an exceedingly high cost. There is much to be gained from building up such redundancy *ex ante*, in a way that may help prevent export restrictions in the first place. A broader range of producer countries would thus work to temper governments’ fears of a “run on export restrictions,” which is what leads to export restrictions in the first place. Trade-dependent countries like Canada, which has played a leading role in related discussions around a global competition policy in the past,^4^ may be in a good position to contribute to such efforts.

## Notes

1 “Switzerland recognized that retaliation against the United States would accomplish nothing” (Irwin, 2017: 169).

2 In keeping with the GTA dataset, I rely on Harmonized System HS2012 product codes.

3 These include exports bans, export quotas, exports taxes, and export licensing schemes.

4 Canada has arguably done the most to advance the conversation around the internationalization of competition policy. See, for instance, Competition Bureau Canada (2015).
